# Equivalent own name bias in autism: An EEG study of the Attentional Blink

**DOI:** 10.3758/s13415-021-00967-w

**Published:** 2021-11-11

**Authors:** Annabel D. Nijhof, Jana von Trott zu Solz, Caroline Catmur, Geoffrey Bird

**Affiliations:** 1 Social, Genetic and Developmental Psychiatry Centre, Institute of Psychiatry, Psychology and Neuroscience, King’s College London, De Crespigny Park, London, SE5 8AF UK; 2 Institute of Medical Psychology, Ludwig-Maximilians-Universität Munich, Goethestrasse 31/I, 80336 Munich, Germany; 3 Department of Psychology, Institute of Psychiatry, Psychology and Neuroscience, King’s College London, De Crespigny Park, London, SE5 8AF UK; 4 Department of Experimental Psychology, University of Oxford, Oxford, OX2 6GG UK

**Keywords:** Attentional Blink, Autism, EEG, Self-bias

## Abstract

The “Attentional Blink” refers to difficulty in detecting the second of two target stimuli presented in rapid temporal succession. Studies have shown that salient target stimuli, such as one’s own name, reduce the magnitude of this effect. Given indications that self-related processing is altered in autism, it is an open question whether this attentional self-bias is reduced in autism. To investigate this, in the current study we utilised an Attentional Blink paradigm involving one’s own and others’ names, in a group of 24 autistic adults, and 22 neurotypical adults, while measuring EEG. In line with previous studies, the Attentional Blink was reduced when the participant’s own name was the second target, with no differences between autistic and neurotypical participants. ERP results show that the effect on the Attentional Blink of one’s own name was reflected in increased N2 and P3 amplitudes, for both autistic and nonautistic individuals. This is the first event-related potential study of own-name processing in the context of the Attentional Blink. The results provide evidence of an intact attentional self-bias in autism, both at the behavioural and neural level.

**Supplementary Information:**

The online version contains supplementary material available at 10.3758/s13415-021-00967-w.

## Introduction

Humans are biased to process preferentially any information that is relevant to ourselves. Previous research demonstrates that preferential processing of self-related stimuli occurs across various cognitive domains, including memory, perception, and attention ( Cunningham & Turk, 2017). This “self-” or “egocentric” bias is present for stimuli that have long been associated with the self, such as one’s own face or name (Bortolon & Raffard, 2018; Wood & Cowan, 1995; Yang et al., 2013), and also for newly self-associated stimuli, such as objects, geometric shapes, and trait adjectives (Cunningham et al., 2008; Sui et al., 2012; Symons & Johnson, 1997). The self-bias is thought to be adaptive not only because it facilitates the creation of a stable sense of self, but it also is thought to benefit adaptive social functioning, as a better model of the self results in more accurate representations of others (Conway et al., 2019; Mitchell, 2009). That is: in order to understand others, humans simulate others’ states in the self. Such simulations allow one to determine which emotions, mental, or other internal states would be experienced if one was in the other’s state and, in turn, these are attributed to the other (Goldman, 2006).

Autism Spectrum Disorder is a neurodevelopmental condition characterized by social communication and interaction difficulties alongside restricted, repetitive behaviours and interests (American Psychiatric Association, 2013). It has been associated with altered self-referential processing, particularly (but not exclusively) in relation to other social agents (Grisdale et al., 2014; Mundy et al., 2010; Nijhof & Bird, 2019; Perrykkad & Hohwy, 2019; Williams, 2010). Observed differences in autistic^1^ individuals’ sense of self would in turn lead to difficulties simulating others’ states in the self and, therefore, may contribute to the socio-cognitive difficulties that are characteristic of autism. In his first description of autism, Kanner (1943) focused on the egocentric nature of autistic behaviour, which would predict an extreme self-bias in autism (Frith & de Vignemont, 2005; Lombardo & Baron-Cohen, 2010). In contrast, later reports indicated that certain aspects of self-preferential processing are in fact diminished in autism (Lyons & Fitzgerald, 2013; Uddin, 2011; Williams, 2010). For example, the response to one’s own name, which is present in neurotypical children around age 5 months (Parise et al., 2010), often is absent or diminished in children who later receive an autism diagnosis. The reduced response to the own name is one of the earliest and best predictors of autism (Miller et al., 2016; Nadig et al., 2007; Werner et al., 2000; Zwaigenbaum et al., 2005).

Despite these findings, studies on the effects of self-relevance in autism are relatively scarce, and results have been inconsistent. Some recent studies indicated that self-bias effects on memory and perception are equivalent in autistic and neurotypical individuals (Lind et al., 2019; Williams et al., 2017). Several researchers have tried to integrate the mixed findings into cognitive models of self-processing in autism. Early proposals suggested that autistic individuals show differences in the psychological but not the physical aspects of self-processing (Uddin, 2011; Williams, 2010). A later suggestion (Williams et al., 2017) is that early-stage (“first-order”) processing of the self (i.e., simply tagging something as self-related, such as a geometric shape in the shape-label matching task; Sui et al., 2012) is intact in autism, whereas a later-stage “second-order” evaluation of the self is affected (e.g., reflecting on the question “Am I a friendly person?”). Two points are important to consider here, however. First, individual levels of self-bias do not appear to generalize across different cognitive domains (Nijhof et al., 2020), suggesting any effects of self-relevance on an individual’s cognitive processing are more fractionated than was previously assumed. Second, neurological differences in processing self-relevant stimuli have been found in the absence of behavioural indices. For example, in three electroencephalography (EEG) studies of processing one’s own name and/or own face (Cygan et al., 2014; Nijhof et al., 2018; Nowicka et al., 2016), the ERP components associated with the processing of self-related stimuli in neurotypical individuals (P3/Parietal Positivity; Knyazev, 2013) were found to be reduced in autistic adults, despite no indication of behavioural differences. Therefore, to gain a better understanding of differential self-processing in autism, studies are needed across all cognitive domains, incorporating behavioural as well as neural measures (Nijhof & Bird, 2019).

It is therefore surprising that, to date, few studies have investigated the processing of self-related information in the attentional domain in autism, although one study did find that autistic individuals showed a reduced influence of self-relevance on the attentional gaze cueing effect (Zhao et al., 2018). One paradigm that has proven particularly useful in investigating the effect of self-bias on attention is the Attentional Blink paradigm. The Attentional Blink is the term used to refer to the difficulty in detecting the second of two target stimuli when these are presented in short temporal succession (Raymond et al., 1992). In studies employing such paradigms, participants are usually asked to identify two target stimuli presented within a rapid serial visual presentation (RSVP) stream of nontarget (distracter) stimuli. When reporting the identified targets, the second target (T2) tends to be missed when presented 200-500 ms after the first target (T1). As attentional resources are being used for processing T1, capacity limitations on these resources are thought to prevent the further processing and conscious perception of T2 (Dux & Marois, 2009; Martens & Wyble, 2010). Interestingly, within the timeframe of the Attentional Blink, particularly salient stimuli, such as familiar faces (Jackson & Raymond, 2006), and emotionally significant images or words (Anderson, 2005; Fox et al., 2005; Ihssen & Keil, 2009; Trippe et al., 2007; Yerys et al., 2014) are detected more often than other stimuli, indicating that less attentional resources are required for such stimuli to be detected. Crucially, it has been shown that the Attentional Blink is also reduced for one’s own name (Shapiro et al., 1997; Tibboel et al., 2013), indicating it is salient enough to cross the threshold for attentional capture, which is also true (but to a lesser extent) for highly familiar names of close others (Nijhof et al., 2020). In this last study, across three experiments with neurotypical samples, no relationship was observed between the self-bias effect on the Attentional Blink and the level of autistic traits. Nevertheless, it could still be the case that such differences would be found when comparing participants with and without an actual autism diagnosis. Furthermore, as argued above, it should be considered that even though behavioural results suggest autistic traits do not relate to the individual level of self-bias in the attentional domain (Nijhof et al., 2020), processing differences may still be observed at the neural level.

The neurocognitive processes underlying the Attentional Blink have been studied extensively using EEG, as event-related potentials (ERPs) allow the study of neural responses time-locked to the presentation of (detected and undetected) target stimuli with high temporal resolution (Craston et al., 2009; Dell’Acqua et al., 2003; Giesbrecht et al., 2007; Jolicœur et al., 2006; Kanske et al., 2013; Kranczioch et al., 2007; MacLeod et al., 2017; Martens et al., 2006; Rolke et al., 2001; Sergent et al., 2005; Vogel & Luck, 2002; Vogel et al., 1998). However, to the best of our knowledge, ERPs in relation to one’s own name in an Attentional Blink paradigm have not yet been investigated, and more generally we are not aware of any ERP studies on the Attentional Blink in autistic individuals. ERP studies in neurotypical individuals suggest that early visual as well as semantic processing is intact irrespective of whether T2 can be reported, as is evident from the fact that missed T2s still elicit early components such as the P1 and N1 (Jolicœur et al., 2006; Sergent et al., 2005; Vogel et al., 1998), as well as the semantic N400 component (Rolke et al., 2001; Vogel et al., 1998; but see Giesbrecht et al., 2007). Divergence of ERP signals when comparing detected and undetected T2s has been observed as early as 200 ms after stimulus onset, at the N2 component (or N2pc in the case of lateralized stimulus presentation), thought to represent attention allocation (Jolicœur et al., 2006; Kranczioch et al., 2007; Sergent et al., 2005). In addition, changes in the centroparietal P3 component for missed targets are among the most reliably reported electrophysiological indicators of the Attentional Blink. Missed T2s, as compared to detected T2s, are reported to either show a delayed P3 peak latency (Martens et al., 2006; Sessa et al., 2007), a reduction in P3 amplitude (Craston et al., 2009; Dell’Acqua et al., 2003; Kanske et al., 2013), or even its complete absence (Kranczioch et al., 2007; Sergent et al., 2005; Vogel & Luck, 2002; Vogel et al., 1998). As the P3 is thought to represent stimulus uptake into working memory (Polich, 2007), its reduction during the Attentional Blink likely conveys that the stimulus is not integrated into working memory and is hence unavailable for conscious report. Salient stimuli, such as one’s own name, that enter awareness more often than other stimuli, might elicit a less diminished (i.e., greater) P3 component at the neurophysiological level than other names when presented at T2. The processing of self-relevant information is associated with increased P3 amplitude (Knyazev, 2013) and with reductions in P3 amplitude in autistic adults (Cygan et al., 2014; Nijhof et al., 2018; Nowicka et al., 2016; although this has not yet been studied in the context of the Attentional Blink). If the P3 amplitude is a reliable correlate of whether or not a stimulus will be detected during the Attentional Blink period, and the P3 is reduced in autistic individuals in response to their own name, the self-bias effect on the Attentional Blink and its neural correlates may be reduced in autistic individuals.

The current study was designed to investigate behavioural as well as neural effects of the self-bias on attention in autism. To this end, an Attentional Blink paradigm was employed while recording participants’ EEG, using T2 stimuli of varying self-relevance. When studying self-preferential processing, it is desirable to disentangle effects of self-relevance and familiarity as far as is possible (Nijhof et al., 2020). Therefore, the participant’s own name (ON), a stranger’s name (SN) and the name of a close other (CN), e.g., a close friend or family member, were used as target stimuli in the Attentional Blink task. Thus, it can be determined whether any enhanced processing of stimuli is due to a true self-referential effect, as reflected by exclusive preferential processing of ON when compared to CN and SN. If, instead, enhanced processing of both ON and CN compared with SN is observed, this enhancement is likely to reflect effects of familiarity or personal significance, rather than self-prioritization.

Behaviourally, we expect to find a classical Attentional Blink effect, represented by a reduction in T2 detection accuracy shortly following the detection of a T1 stimulus, in both the autistic and nonautistic group. We hypothesize that the reduction of the Attentional Blink in the own name condition would be less pronounced in autistic compared to neurotypical individuals. Regarding the ERPs, we expect the P3, and possibly the N2 component, to reflect the salience of the T2 stimulus and therefore to be of larger amplitude for the ON than for the CN and SN, and potentially larger for the CN than for the SN, in line with the behavioural hypotheses. Finally, the hypothesized enhancement of the P3 for one’s own name is expected to be diminished in autism.

## Methods

### Participants

Initially, 26 autistic adults and 25 neurotypical participants were tested. However, two of the autistic participants and three participants from the neurotypical group were excluded due to low (<50% correct) T1 detection rates (Autism: N = 1, Neurotypical: N = 2) or technical failure (N = 1 for both groups). The final behavioural sample therefore consisted of 24 autistic participants (13 males) and 22 neurotypical participants (13 males). Furthermore, due to an insufficient number of T1-correct, artifact-free trials, EEG data for three autistic and two neurotypical participants could not be analysed. Table 1 provides an overview of the demographics of the final participant sample for ERP analysis (see Supplementary Table 1 for demographics of the full behavioural sample).

**Table 1:** Demographics for the two groups (EEG sample only)

	Autism (N = 21, 11 males) *M (SD)*	Neurotypical (N = 20, 11 males) *M (SD)*	T, *p* values
Age (years)	29.9 (6.0)	30.9 (7.8)	0.48, p = .63
AQ	36.0 (6.8)	14.4 (7.5)	9.6, p < .001
WASI	112.1 (11.8)	113.5 (12.0)	0.36, p = .72

AQ = Autism Spectrum Quotient, WASI = Wechsler Abbreviated Scale of Intelligence

All participants had normal or corrected-to-normal vision. Autistic participants were recruited from an existing volunteer database and neurotypical participants via an institutional research volunteer recruitment newsletter and webpage, as well as social media advertisements. Autistic volunteers were only included if they had a formal diagnosis of autism, Asperger’s syndrome (DSM-IV), or Autism Spectrum Disorder (DSM-5). Participants’ diagnosis was verified with the Autism Diagnostic Observation Schedule (ADOS-2, Module 4; Lord et al., 2012), performed by a trained psychologist. Participants in both groups completed the 50-item Autism Spectrum Quotient (AQ) (Baron-Cohen, Wheelwright, Skinner, Martin, & Clubley, 2001)—a widely-used self-report measure of autistic traits. In addition, an estimate of IQ scores of all participants was obtained with the Vocabulary and Matrix Reasoning subtests of the Wechsler Abbreviated Scale of Intelligence (WASI-II; Wechsler, 2011). All participants gave written, informed consent prior to the study and were reimbursed financially. The study was approved by the university’s ethics committee under the reference number HR-17/18-5537.

### Procedure

Participants took part in a single session of approximately 150 minutes. After the EEG was set up, EEG activity was recorded during three task blocks of approximately 30 minutes each. After the EEG session, participants completed the AQ questionnaire and the WASI-II if no IQ estimate was already available. For the autistic participants, if the ADOS had not yet been performed, this was completed in a separate test session together with the WASI-II.

### Name stimuli

Prior to the experiment, participants were asked to provide their own first name and that of another person close to them (*“What is the first name of someone very close to you? (for example, a family member or close friend)”*). For the stranger’s name, as well as the distractor names, we selected 40 popular English names from two databases (BabyCentre, 2000; UK Office for National Statistics, 2014). To avoid effects of personal significance in the stranger’s name condition or for distractor names, we asked participants to highlight those names on the list of 40 names that they associated with people they were personally familiar with and excluded these from the paradigm on an individual basis. Of the remaining names, one name similar in length to the CN was chosen as the target name for the SN condition. On average, names in all three conditions were between 5 and 6 digits long (ON: 5.4, CN: 5.3, SN: 5.6), with no significant differences between either groups (F(1, 44) = 0.19, *p* = 0.67, η_p_^2^ < 0.01) or conditions (F(2, 88) = 0.51, *p* = 0.54, η_p_^2^ = 0.01).

As subjective self-other closeness ratings have been shown to relate to neural responses to others (Courtney & Meyer, 2020), after task completion, participants were asked to rate how close they feel to their chosen close other and assigned stranger, by completing two “Inclusion of Other in the Self” scales. These are visual representations of two circles overlapping to various degrees, representative of the degree of closeness felt between the self and the other person (Aron et al., 1992). These data revealed participants felt significantly closer to the CN than to the SN (t(45) = 16.94, *p* < 0.001, d = 2.44; CN: M: 5.3 (SD: 1.6), SN: M: 1.4 (SD: 0.7)), and the difference between CN and SN was not significantly different between groups (t(44) = 0.10, *p* = 0.92, d = 0.03), nor were the absolute levels of felt closeness (CN: t(44) = 0.22, *p* = 0.83, d = 0.07; SN: t(44) = 0.28, *p* = 0.78, d = 0.09).

### Task

**Fig. 1:**
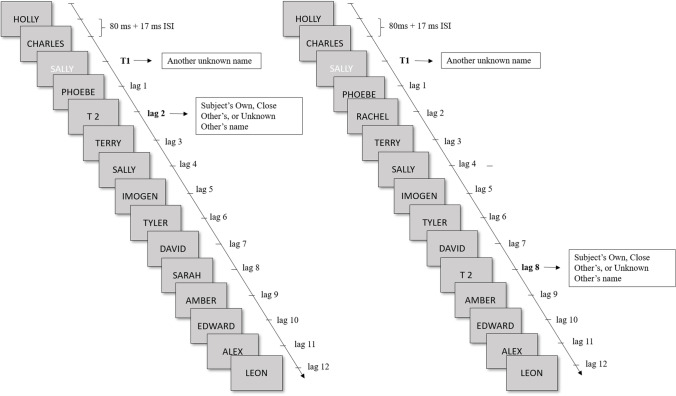
Visualisation of the Attentional Blink paradigm, with examples for the second target (T2) at Lag 2 and Lag 8. Note that the first target (T1) could be presented at position 3 (as shown here), 4, or 5

The Attentional Blink task was presented using PsychoPy version 1.85.2 (Peirce, 2007) on a 17-inch LCD monitor. RSVP streams of 15 first names were presented to participants (see Fig. 1). The different T2 conditions (ON, CN, SN) were presented in three different blocks, the order of which was counterbalanced between participants. Following a red fixation cross presented centrally for 1,000 ms, all 15 names were presented for 80 ms, with an inter-stimulus interval of 17 milliseconds. All stimuli were presented centrally on the screen in capitalised 40-point Arial, on a light grey background. Except for the T1, which was printed in white, all of the other 14 names were printed in black. T1 was presented either at the third, fourth or fifth position in the stream. The T2 name was presented at two different lags: Lag 2 and Lag 8, where the number corresponds to the number of stimulus presentations after T1 that T2 was presented. While Lag 2 stimuli are situated within the attentional blink, stimuli presented at Lag 8 represent a period outside the blink. These lags are sufficient to give an approximation of the magnitude of the AB effect, as this is determined by the difference between performance during and outside the blink (Martens & Wyble, 2010). Furthermore, the current paradigm is based on a previous study (Nijhof et al., 2020), which included a control condition in which no T1 was reported, as well as T2 targets at lags 1, 2, 5, and 8, in which the AB was observed. After the RSVP stream had finished, participants were prompted with two questions: 1) “What was the white word?”; 2) “Was [T2 name] present or not present?” To answer the first question, participants typed the name on the keyboard; to answer the second, they pressed either the ‘c’ key (present) or ‘n’ key (not present).

The first block was preceded by ten practice trials. Subsequently, T2 was present at Lag 2 and at Lag 8 on 25% of trials each and was absent on the remaining 50% of trials. In an attempt to keep task duration as short as possible while still acquiring sufficient trials for ERP analysis, the task was programmed to stop once at least 22 trials were obtained on which the T2 was detected at Lag 2, as well as at least 22 on which it was missed. In addition, unless these numbers had been reached, the experiment would continue for a maximum of 360 trials per block (180 T2 present, 180 T2 absent). This strategy led to an average number of 318 trials per block per participant.

### Behavioural data analysis

First, the number of correct T1 detections was calculated for each condition. Minor spelling mistakes were tolerated (e.g., Lacy for Lacey) if the answer was still recognisable as the correct name. Using this criterion, all incorrect responses were manually checked by two independent raters, who showed an agreement rate of 88.4%. Any disagreements were resolved through discussion. Second, a 3 x 2 x 2 repeated-measures ANOVA was performed to test whether T1 detection accuracy differed by Condition (ON/CN/SN), Lag (2/8) or Group. Third, we calculated the proportion of “T1 correct” trials on which T2 was also correctly detected, for each lag and condition. The proportion of T2 trials correctly detected (given correct T1 detection) served as the dependent variable in a similar 3 x 2 x 2 repeated-measures ANOVA, again including factors of Condition (ON/CN/SN), Lag (2/8), and Group.

### EEG recording and data analysis

EEG data were acquired from a 64-channel DC-coupled recording system (Brain Products, Gilching, Germany) with a ground electrode at AFz, and a reference electrode at FCz. The sampling rate was 500 Hz, and we aimed to keep impedances below 15 kΩ throughout the recording session.

Offline, data were re-referenced against the average, after which FCz was included as a regular electrode. A high-pass filter of 0.5 Hz (12 dB/oct), a low-pass filter of 30 Hz (12 dB/oct), and a notch filter at 50 Hz were applied. Any bad channels were interpolated, and ocular correction was applied through Independent Component Analysis, using Fp2 as the vertical eye channel and the difference between AF7 and AF8 as the horizontal eye channel. Subsequently, the outer six channels (Fp1, Fp2, TP9, TP10, FT9, FT10) were removed. Next, data were manually inspected for artifacts, and any applied DC-offset corrections were removed. Data for trials on which T1 was correctly detected were then segmented by block (i.e., by condition) using factors *Lag* and *T2 Detection Accuracy (detected/missed)*. The resulting epochs of 1,100 ms were computed with an onset time-locked to the T2 stimulus, and a baseline of 100 ms before T2. Subsequently, semiautomatic artifact rejection was applied to all segments, and those segments containing voltage steps of more than 50 μV/ms, amplitudes of ±100 μV, or low wave activity under 0.5 μV/100 ms were rejected. This resulted in an average of 61 remaining segments per condition for Lag 2 (correctly detected and missed trials collapsed together, as the number of detected/missed trials alone was not sufficient to analyse these separately), and 49 for Lag 8 (correctly detected only). Finally, baseline correction was performed using the 100-ms preonset interval, and segments were averaged per condition and participant.

We defined components and analyses on the basis of earlier ERP studies of the Attentional Blink (Craston et al., 2009; Dell’Acqua et al., 2003; Jolicœur et al., 2006; Kranczioch et al., 2007; Martens et al., 2006; Vogel & Luck, 2002; Vogel et al., 1998), as well as visual inspection of the grand-average ERPs and scalp topography across conditions. ERPs for Lag 8 showed clear N2 and P3 components, whereas P3 was visually less pronounced at Lag 2, except for the ON condition.

Correctly detected T2s at Lag 8 were analysed initially, as these would provide an index of relatively unaffected T2 processing, with a high number of trials. Time windows for which we exported mean ERP amplitudes for further statistical analyses included a negative component 250-310 ms after T2 onset (henceforth N2) and a positive component 320-380 ms after T2, resembling a P3 peak (henceforth P3). Analyses were focused on left- and right-lateralized parieto-occipital electrodes (P3, P4, P7, P8, PO3, PO4, PO7, PO8, O1, O2), as the topography showed parieto-occipital activations for both N2 and P3, and as electrophysiological activations related to both the AB and visual name perception were previously most reliably reported in parietal locations (Craston et al., 2009; Dell’Acqua et al., 2003; Tacikowski et al., 2014; Vogel & Luck, 2002). Electrodes were combined into a left-lateralized cluster (P3, P7, PO3, PO7, O1) and a right-lateralized cluster (P4, P8, PO4, PO8, O2).

### Statistical reporting

Both behavioural and extracted ERP data were analysed using IBM SPSS version 24. Where data are analysed using ANOVA, partial eta squared (η_p_^2^) values are reported as a measure of effect size; Cohen’s d is reported for t-tests (Cohen, 1988). In cases where the assumption of sphericity was violated, a Greenhouse-Geisser correction was applied, and corrected values are reported. Furthermore, it is reported if follow-up comparisons did not survive Bonferroni correction for multiple comparisons.

Because the primary goal was to investigate the presence or absence of group differences in self-bias, results of repeated-measures ANOVAs also were analysed within a Bayesian framework using JASP (https://jasp-stats.org; JASP Team, 2016) to examine the strength of the evidence in favour of the null and alternative hypotheses. A Bayes Factor (BF10) approaching zero indicates that the data provide more evidence in favour of the null hypothesis (H0) than the alternative hypothesis (H1), a value of 1 indicates that H0 and H1 are equally likely given the data, and values above 1 indicate greater support for H1. By convention, values below one third and above 3 are taken as evidence in favour of H0 and H1, respectively, whereas values between these values are judged to provide insufficient evidence to favour either hypothesis.

## Results

### Behavioural data

Correct T1 detection was high overall (84.3% on average), and a 3 (Condition) x 2 (Lag) x 2 (Group) repeated-measures ANOVA did not reveal any significant effects: T1 detection did not differ significantly between groups (F(1, 44) = 1.70, *p* = 0.20, η_p_^2^ = 0.04, BF10 = 0.28), lags (F(1, 44) = 2.02, *p* = 0.16, η_p_^2^ = 0.04, BF10 = 0.09), or between the three T2 conditions (F(2, 88) = 0.25, *p* = 0.71, η_p_^2^ < 0.01, BF10 = 0.02), and all interaction effects were non-significant (all *p* > 0.12, all BF10 < 0.31).

**Fig. 2:**
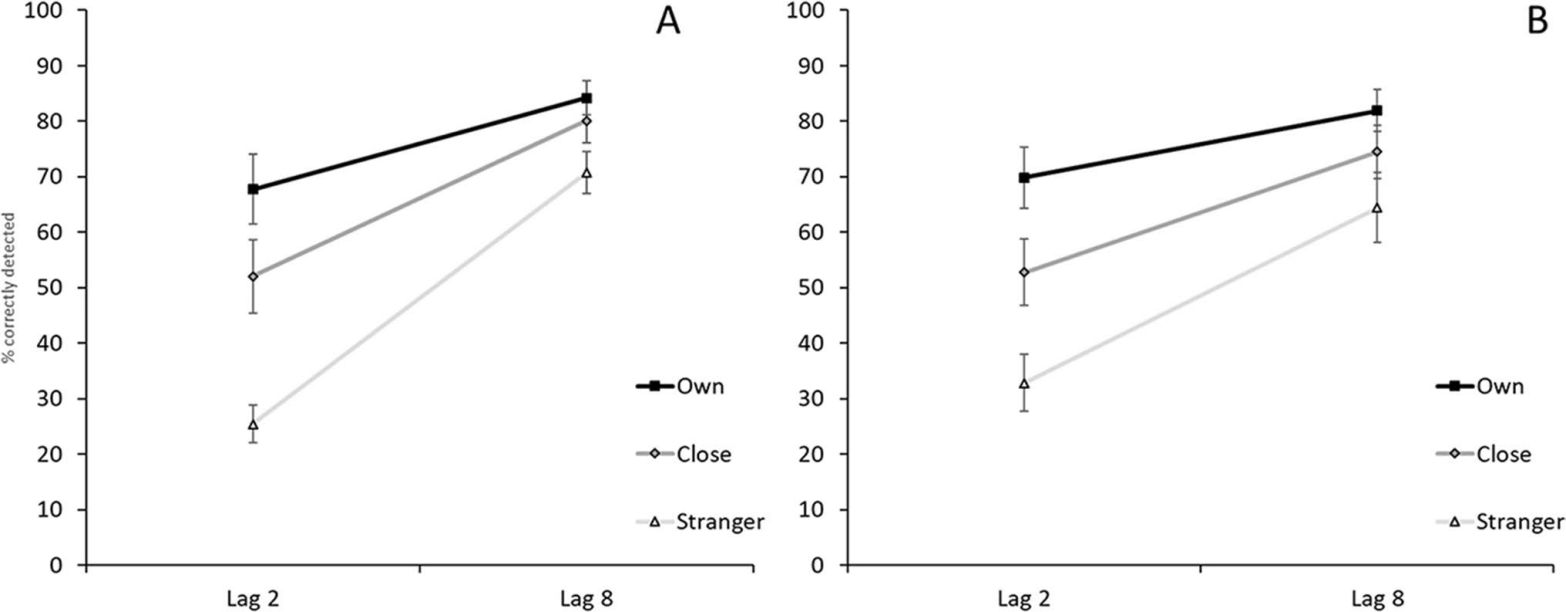
Mean T2 detection rates for the two different lags (given correct detection of T1), ± 1 standard error of the mean. Neurotypical group (**A**); Autism group (**B**). T1 was a stranger's name, T2 was either the participant’s own name, the name of somebody close to them, or the name of a stranger

Behavioural results for T2 detection for both groups are displayed in Fig. 2. For the analysis of correct T2 detections (given T1 was correct), the 3 (Condition) x 2 (Lag) x 2 (Group) repeated-measures ANOVA showed a significant main effect of Condition (F(2, 88) = 50.86, *p* < 0.001, η_p_^2^ = 0.54, BF10 = 1.40*10^14^). T2 detection of the ON was better than for either CN or SN conditions (both *p* < 0.001), and better in the CN than the SN condition (*p* < 0.001). Furthermore, the main effect of Lag was significant (F(1, 44) = 165.17, *p* < 0.001, η_p_^2^ = 0.79, BF10 = 1.40*10^14^): T2 detection was worse at Lag 2 than at Lag 8 (*p* < 0.001), indicative of the typical attentional blink effect. In addition, the data showed a significant Condition x Lag interaction (F(2, 88) = 24.11, *p* < 0.001, η_p_^2^ = 0.35, BF10 = 4.49*10^3^): the difference between Lag 2 and Lag 8 was significantly smaller (indicating a smaller attentional blink) in the ON than the CN condition (t (45) = 3.78, *p* < 0.001, d = 0.56) and the SN condition (t (45) = 6.07, *p* < 0.001, d = 0.90), as well as smaller for CN than SN (t (45) = 3.71, *p* = 0.001, d = 0.55).

There was no significant main effect of Group (F(1, 44) = 0.01, p = .91, η_p_^2^ < .01, BF10 = 0.29), nor was the three-way interaction between Condition, Lag and Group significant (F(2, 88) = 0.99, p = .37, η_p_^2^ = 0.02, BF10 = 0.08). The difference between Lag 2 and 8 between ON and CN did not significantly differ between the two groups (t (44) = 0.35, p = 0.73, BF10 = 0.31).

The interaction between Lag and Group showed an effect (albeit nonsignificant and not supported by the Bayesian analysis) that was driven by slightly better performance at Lag 2 and slightly worse performance at Lag 8 in the autism group than in the neurotypical group (F(1, 44) = 4.06, *p* = 0.05, η_p_^2^ = 0.08, BF10 = 0.79).

### ERP data

**Fig. 3:**
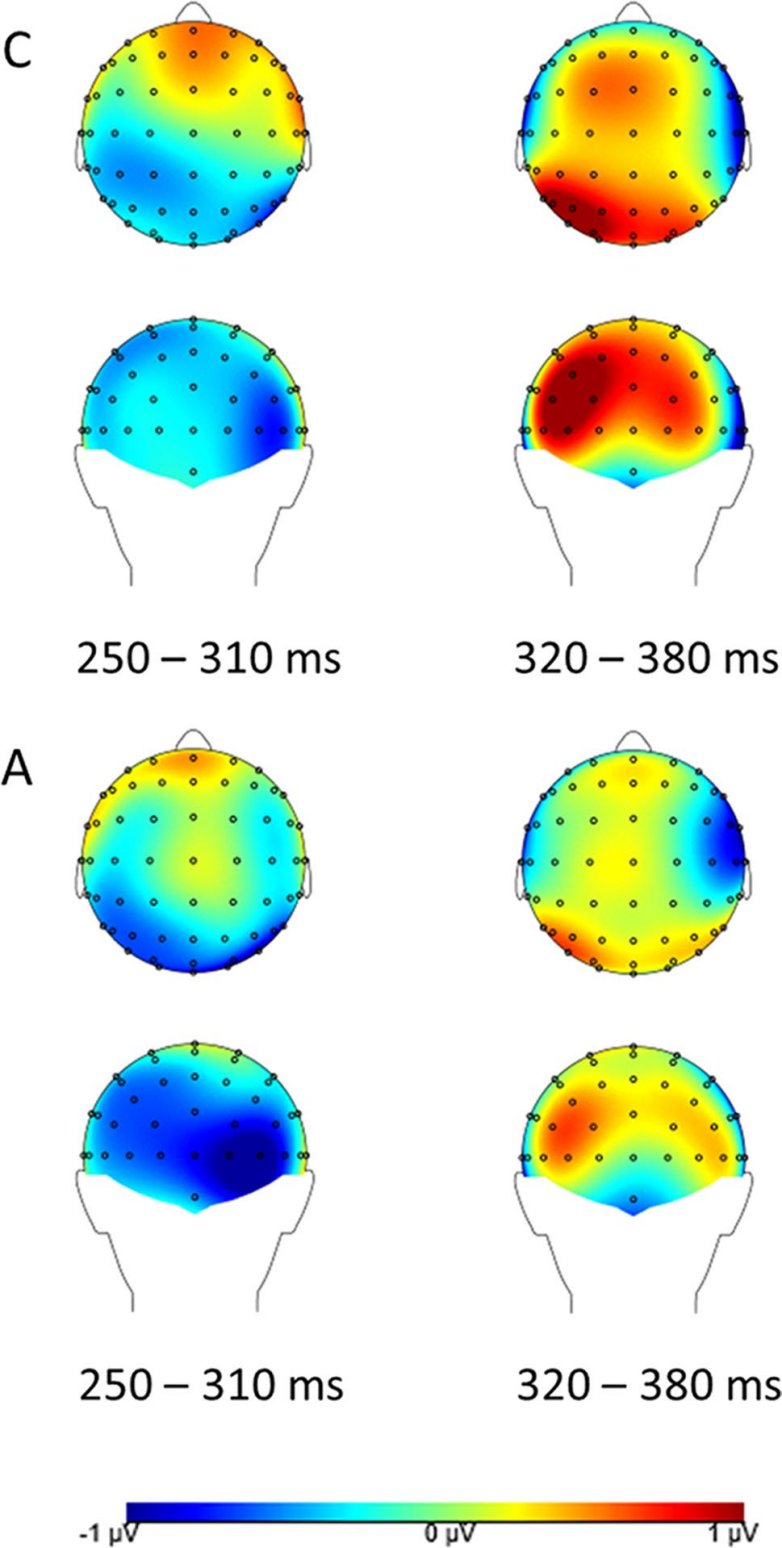
Difference topography (own minus close other’s name) for both groups for correctly detected T2s presented at Lag 8. Left: N2 time window. Right: P3 time window. Top: Neurotypical group (**C**). Bottom: Autism group (**A**)

**Fig. 4:**
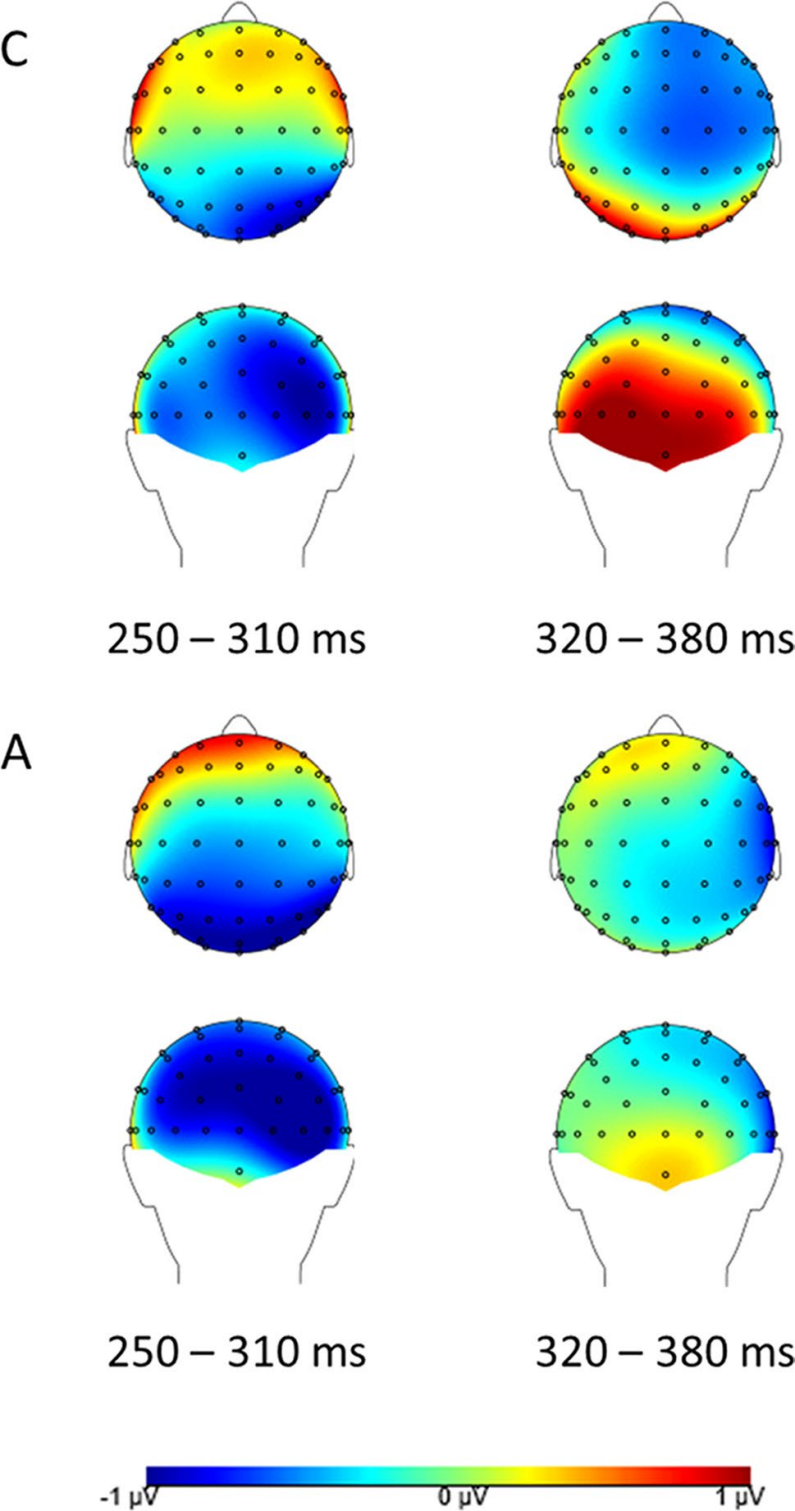
Difference topography (own minus close other’s name) for both groups for (detected and missed) T2s presented at Lag 2. Left: N2 time window. Right: P3 time window. Top: Neurotypical group (**C**). Bottom: Autism group (**A**)

Difference topography plots (own minus close other’s name) across both groups for T2s presented at Lag 8 and Lag 2 are presented in Figs. 3 and 4 respectively (note that for Lag 2 data correct detections and misses are analysed together, whereas Lag 8 data consist of correct detections only).

#### Lag 8

**Fig. 5:**
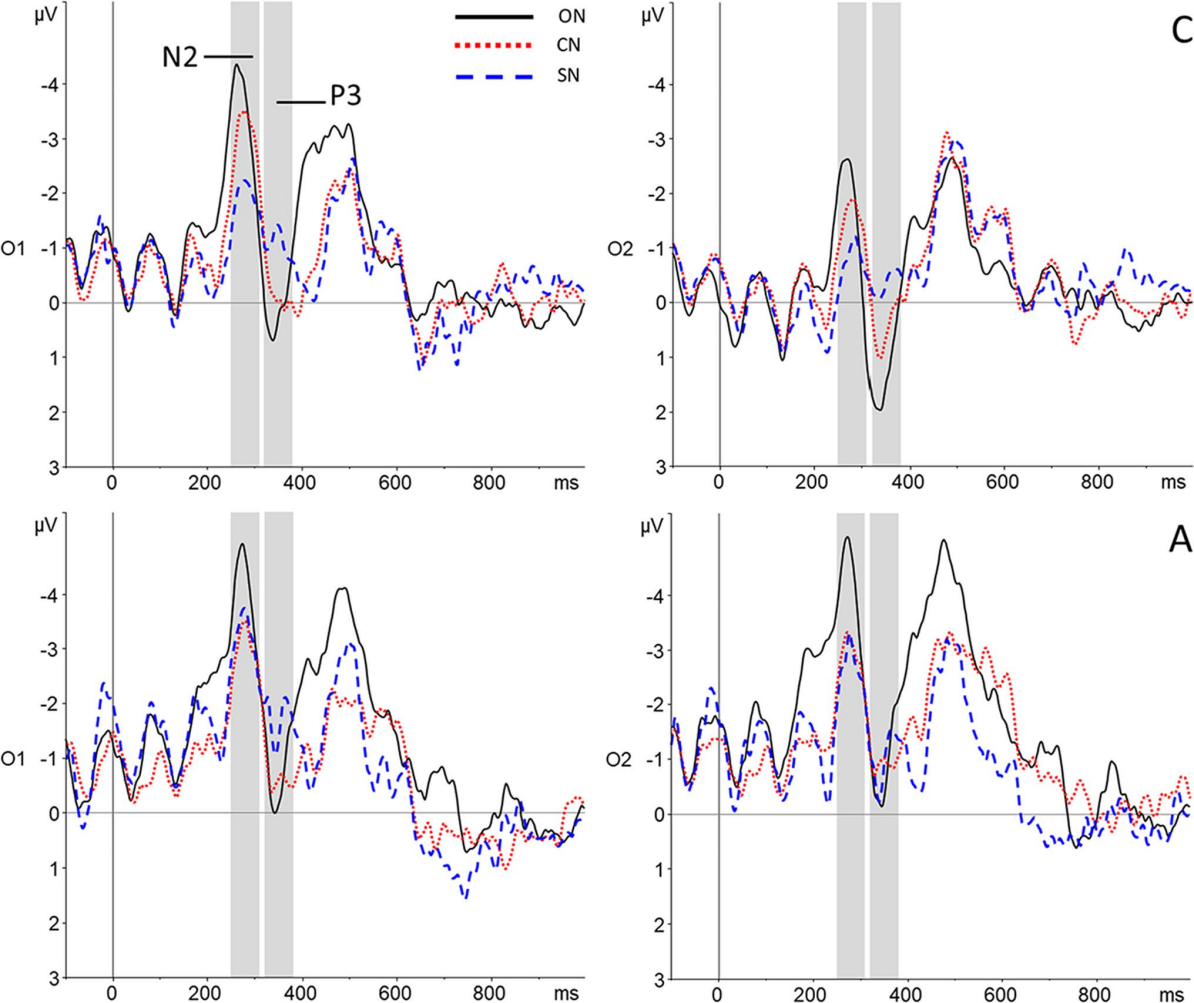
Grand average waveforms for two electrodes (O1 and O2) included in the N2 and P3 analyses for correctly detected T2s presented at Lag 8. ON = Participant’s own name; CN = Close other’s name; SN = Stranger’s name. Top: Neurotypical group (**C**). Bottom: Autism group (**A**)

Grand average waveforms for Lag 8 are displayed in Fig. 5. For one participant from the autism group, there were insufficient correctly-detected trials in the Lag 8-SN condition to calculate a reliable average. Therefore, Lag 8 data were analysed for 20 participants per group.

##### N2 analysis

A 3 x 2 x 2 ANOVA with within-subjects factors Condition (ON, CN, SN) and Laterality (left cluster, right cluster), and between-subjects factor Group (Neurotypical group, Autism group) revealed a significant main effect of Condition (F(2, 76) = 5.06, *p* = 0.009, η_p_^2^ = 0.12, BF10 = 17.99). The N2 amplitude was significantly larger in the ON than the SN condition (*p* = 0.003, d = 0.52). The difference between ON and CN (*p* = 0.19, d = 0.21), and between CN and SN (*p* = 0.08, d = 0.29) did not reach significance. Further, the main effect of Laterality was significant (F(1, 38) = 31.32, *p* < 0.001, η_p_^2^ = 0.45, BF10 = 3.01*10^7^), with a larger N2 amplitude in the left-lateralized cluster. No other effects were significant, and a Bayesian ANOVA provided substantial evidence that there was no significant Condition x Group interaction (BF10 = 0.12).

##### P3 analysis

The ANOVA with within-subjects factors Condition (ON, CN, SN) and Laterality (left cluster, right cluster), and between-subjects factor Group (Neurotypical group, Autism group) showed a significant main effect of Condition (F(2, 76) = 4.93, *p* = 0.01, η_p_^2^ = 0.12, BF10 = 32.47): the P3 amplitude was significantly larger in the ON than the SN condition (*p* = 0.001, d = 0.56). The difference between ON and CN did not reach significance (*p* = 0.08, d = 0.27), nor did that between CN and SN (*p* = 0.27, d = 0.18). The main effect of Laterality also was significant (F(1, 38) = 26.60, *p* < 0.001, η_p_^2^ = 0.41, BF10 = 28.89*10^3^), with a larger P3 amplitude in the right cluster. Neither the main effect of Group, nor any of its interaction effects, were significant (all *p* > 0.29). A Bayesian ANOVA revealed substantial evidence that Group did not interact with the main effect of Condition (BF10 = 0.10).

There was, however, a significant Condition x Laterality interaction effect (F(2, 76) = 4.29, *p* = 0.02, η_p_^2^ = 0.10, although BF10 = 0.20). The effect of Condition was significant in the left cluster (F(2, 76) = 7.32, *p* = 0.001, η_p_^2^ = 0.16), with the difference between both ON and CN (*p* = 0.03, d = 0.36), and between ON and SN (*p* < 0.001, d = 0.68), being significant, although the difference between ON and CN did not survive Bonferroni correction. There was no significant difference between CN and SN (*p* = 0.16, d = 0.23). In the right cluster, the effect of Condition was not significant (F(2, 76) = 1.89, *p* = 0.16, η_p_^2^ = 0.05).

#### Lag 2

**Fig. 6:**
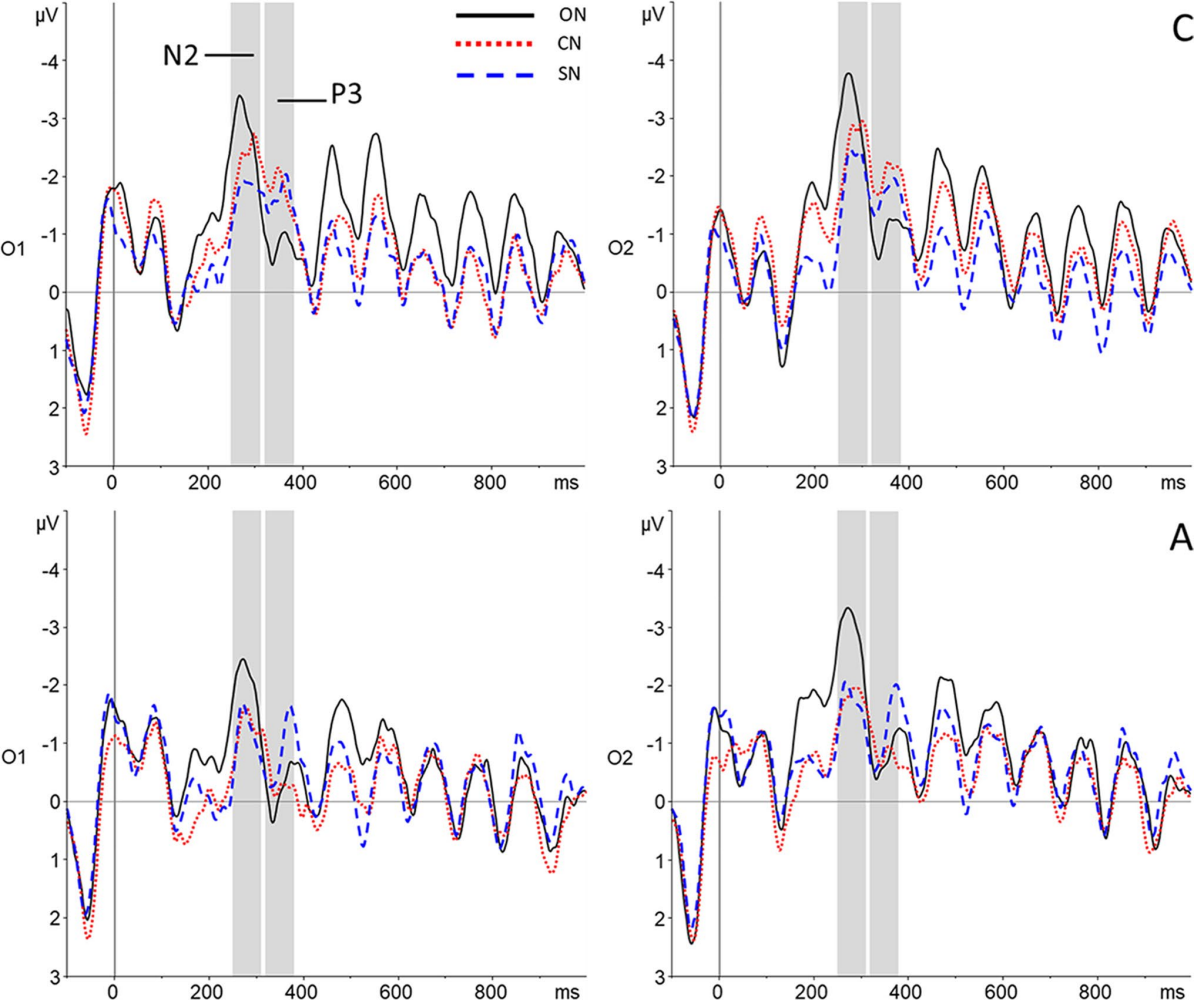
Grand average waveforms for two electrodes (O1 and O2) included in the N2 and P3 analyses for (detected and missed) T2s presented at Lag 2. ON = Participant’s own name; CN = Close other’s name; SN = Stranger’s name. Top: Neurotypical group (**C**). Bottom: Autism group (**A**)

Grand average waveforms and topographical plots for Lag 2 are displayed in Fig. 6.

##### N2 analysis

A 3 x 2 x 2 ANOVA with within-subjects factors Condition (ON, CN, SN) and Laterality (left cluster, right cluster), and between-subjects factor Group (Neurotypical group, Autism group) showed a significant main effect of Condition (F(2, 78) = 10.46, *p* < 0.001, η_p_^2^ = 0.21, BF10 = 14.12*10^4^). The N2 amplitude was significantly larger in the ON condition than in both the CN (*p* = 0.01, d = 0.43) and the SN condition (*p* < 0.001, d = 0.84). The difference between the CN and SN condition was not significant (*p* = 0.28, d = 0.17). The main effect of Laterality was also significant (F(1, 39) = 13.59, *p* = 0.001, η_p_^2^ = 0.26, BF10 = 36.27), as the N2 amplitude was larger in the right cluster than in the left cluster. Although the mean amplitude in the neurotypical group was larger than in the autism group, this effect was not significant (F(1, 39) = 3.10, *p* = 0.09, η_p_^2^ = 0.07, BF10 = 0.42), nor were any of the interaction effects (all *p* > 0.19). Finally, a Bayesian ANOVA showed substantial evidence that Group did not interact with the main effect of Condition (BF10 = 0.13).

##### P3 analysis

The ANOVA with within-subjects factors Condition (ON, CN, SN) and Laterality (left cluster, right cluster), and between-subjects factor Group (Neurotypical group, Autism group) showed a main effect of condition that was not significant (F(2, 78) = 2.96, *p* = 0.06, η_p_^2^ = 0.07, BF10 = 2.26). Planned pairwise comparisons with the ON condition did, however, suggest a similar pattern as for the N2, with the amplitude being larger in the ON condition than the SN condition (*p* = 0.02, d = 0.37), although not surviving Bonferroni correction. The difference between ON and CN was not significant (*p* = 0.09, d = 0.25). The main effect of Laterality was also not significant (F(1, 39) = 3.43, *p* = 0.07, η_p_^2^ = 0.08, BF10 = 0.49), although amplitudes were somewhat larger in the left cluster. Finally, the main effect of Group was significant (F(1, 39) = 6.62, *p* = 0.01, η_p_^2^ = 0.15, BF10 = 3.37), as P3 amplitude was larger in the autism group. Again, none of the interaction effects were found to be significant (all *p* > 0.10). A Bayesian ANOVA provided no evidence for either the presence or absence of a Condition x Group interaction (BF10 = 1.75).

## Discussion

In this study, we investigated attenuation of the Attentional Blink when one’s own name (compared with other names) is presented as the second target, comparing this effect between a group of adults with autism and a group of neurotypical adults. We tested hypotheses of an atypical attentional self-bias in autism, both at the behavioural and at the neural level. To the best of our knowledge, this is both the first ERP study of the Attentional Blink in autism, and of the Attentional Blink where names are presented as targets. As hypothesised, we found evidence for a reduction of the Attentional Blink by the participant’s own name and, to a lesser extent, a close other’s name, both in terms of T2 detection rate and as reflected in N2 and P3 amplitudes. These reductions of the Attentional Blink were not diminished in autism, neither at the behavioural, nor at the neural level, with data not consistent with theories of a reduced self-bias in autism (Nijhof & Bird, 2019; Perrykkad & Hohwy, 2019; Uddin, 2011; Williams, 2010).

More specifically, behavioural results replicated those of our previous study (Nijhof et al., 2020), showing a clear Attentional Blink for all names at Lag 2, which was significantly reduced for the own name compared with both other types of name, and for the close other’s name compared with the stranger’s name, indicative of both self-referential and familiarity effects. The lack of any group effects extends earlier findings showing no correlation between the self-bias in the Attentional Blink task and autistic traits in neurotypical samples (Nijhof et al., 2020).

Existing behavioural studies of the Attentional Blink in autism have shown reduced modulation of the Attentional Blink by salient emotional stimuli in autistic individuals (Corden et al., 2008; Gaigg & Bowler, 2009; Yerys et al., 2014). However, this attenuated modulation of the Attentional Blink may not directly relate to attentional differences in autism: due to the emotional nature of the stimuli, it may be better explained by the increased presence of alexithymia in autistic individuals, as alexithymia predicts emotion processing difficulties (Bird & Cook, 2013). In contrast, studies employing neutral letter-string paradigms found no group differences in the size of the Attentional Blink generally (Amirault et al., 2009; Rinehart et al., 2010), in line with the absence of any behavioural group differences in the current study. The study by Amirault et al. (2009) did report that the Attentional Blink may be prolonged in autism. Here, we only found a trend in this direction, illustrated by the Group x Lag interaction in the behavioural data. Taken together, these results suggest that the temporal capacity-limit on selective attentional resources thought to underlie the Attentional Blink (Dux & Marois, 2009; Martens & Wyble, 2010) is similar between autistic and neurotypical individuals. This study thus adds to the broader literature of studies on (visuospatial) attention in autism, which to date has shown inconsistent findings (Allen & Courchesne, 2001; Bird et al., 2006; Grubb et al., 2013a, b; Ronconi et al., 2018).

Regarding the ERP findings, scalp topography showed the N2 and P3 components known to be involved in the Attentional Blink most clearly at parieto-occipital sites. For the N2 component, this is in line with earlier findings (Jolicœur et al., 2006; Kranczioch et al., 2007; Sergent et al., 2005), but for the P3, previous studies focused on more (centro-)parietal sites (Craston et al., 2009; Dell’Acqua et al., 2003; MacLeod et al., 2017; Vogel & Luck, 2002). Because this is the first ERP study of name processing in an Attentional Blink context, our finding of a somewhat more posterior topography warrants replication in future studies. Generally, across the different lags and components, a pattern of greater amplitudes for increasing familiarity of the T2 name was observed.

At Lag 2, the N2 component, thought to reflect early attention allocation (Jolicœur et al., 2006), showed a clear self-specific effect: amplitudes were larger specifically when one’s own name was the second target. The difference in the number of detected versus missed T2s between conditions did not allow separate analysis of detected and missed T2s, and so we cannot determine whether amplitudes were specifically larger for detected trials in line with previous ERP studies of the Attentional Blink. However, the difference between conditions does show that self-relevance of the T2 affects attentional allocation (Sergent et al., 2005), in line with the behavioural data. Such early attentional capture by self-related stimuli has also previously been shown using different task paradigms (Alexopoulos et al., 2012; Pfister et al., 2012).

P3 amplitude at Lag 2 showed a similar self-advantage, although here, the numerical difference between the participant’s own name and the close other’s name was not statistically significant. Although the name of the close other did not elicit amplitudes that were significantly different in size from either the participant’s own name or the stranger’s name, findings for this condition do appear to reflect a gradual increase in ERP amplitude with increasing familiarity, in line with the behavioural results. In fact, the results for the close other’s name could even be interpreted as an effect of self-relatedness, when considering that one’s identity is strongly shaped through our interactions with (close) others (Gallagher, 2013). As the P3 component represents the updating of stimuli into working memory (Polich, 2007), these findings suggest that the self-bias in the context of the Attentional Blink acts both on early attentional and later working memory processing. In addition, the P3 amplitude at this lag was larger for autistic individuals irrespective of condition. In neurotypicals, the P3 has previously been shown to habituate in response to repeated auditory, visual, and somatosensory stimuli (Nakata et al., 2015; Romero & Polich, 1996). Given the high number of trial repetitions, and reports of reduced visual habituation in autism (Jamal et al., 2020; Kleinhans et al., 2009; Vivanti et al., 2018; Webb et al., 2010), the larger P3 amplitude in the autism group might represent reduced habituation in response to the repeated visual stimulation.

For comparison, correctly detected names at Lag 8 also were analysed. At this lag, more attentional resources are available, and in paradigms where attentional resources are not limited, P3 amplitude has been consistently associated with own-name processing (Knyazev, 2013). At Lag 8, the only significant differences in both N2 and P3 amplitude were between the participant’s own name and the stranger’s name. However, for the P3, despite greater amplitudes overall in the right-lateralised cluster, differential amplitudes for increasing levels of familiarity were found in the left-lateralised cluster. This is similar to findings from studies on face processing, where participants show an increased bilateral, rather than right-lateralised, response to their own or familiar faces (Campbell et al., 2020; Keyes & Brady, 2010).

The results of this study add to a growing body of evidence indicating equivalent self-bias effects on cognition in autism (Lind et al., 2019; Nijhof et al., 2020; Williams et al., 2017), and thus shed further light on the debate over which aspects of self-related processing are, and which are not, atypical in autism. It should be noted that previous studies that did show neural differences in own-name processing between adults with and without autism (Cygan et al., 2014; Nijhof et al., 2018; Nowicka et al., 2016), all did so at relatively late stages of cognitive processing (late P3/late parietal positivity). This is in line with the suggestion that early-stage self-processing in autism is unaffected, with differences only appearing at later stages of cognitive processing (Nijhof & Bird, 2019; Williams et al., 2017), but more research on this later-stage self-processing is needed.

Since this was the first ERP study of names presented during the Attentional Blink, it warrants future replication in larger samples, especially because the groups for whom ERPs could be analysed were relatively small. Even so, it is clear that the pattern of results for the different conditions was highly similar between groups (as also suggested by the Bayesian analyses), emphasizing the presence of attentional self-bias in autism, and results are consistent with behavioural results from earlier studies with larger samples which investigated effects of autistic traits in neurotypical samples (Nijhof et al., 2020). A further limitation beside sample size is that, as mentioned, ERP results for Lag 2 include a mix of detected and missed T2s. Given the high detection rate for the own name, and the low detection rate for the stranger’s name, it was not feasible to collect a sufficient number of trials for each condition and participant, since the experiment was already lengthy and tiresome to complete for participants. The consequences of this do not impact the conclusions relating to self-specificity of the N2 and P3 components, but mean that one cannot conclude, for example, that condition effects on the P3 component reflect entry to into working memory so that stimuli are available for conscious report. Thus, this limitation does not detract from the main finding that autistic and neurotypical individuals show equivalent self-name processing at both behavioural and neurophysiological levels. Finally, it should be noted that the high number of trial repetitions may have led not only to habituation effects, but also to experimental familiarity with the stranger’s name, potentially reducing the strength of any differences between conditions.

In summary, our study indicates that attentional self-bias is not altered in autism: processing of self- and other-related stimuli as second targets in an Attentional Blink paradigm yielded no differences in detection accuracy between autistic and neurotypical individuals. This was underlined by the neural findings, as both under limited attentional resources (Lag 2, during the blink) and when more attentional resources were available (Lag 8), ERP components for both groups provided evidence of effects of self-relevance and familiarity.

## Supplementary Information

ESM 1 (DOCX 28 kb)

## Footnotes

1 To respect the wishes of autistic individuals and to use standard scientific parlance, we will henceforth use the term “autistic,” a term endorsed by many individuals with ASD, as well as language preferred by clinical professionals, e.g., “individuals with autism” (Kenny et al., 2016).
