# Supplementary material for: Equivalent own name bias in autism: An EEG study of the Attentional Blink
Source: Cogn Affect Behav Neurosci. 2021 Nov 11;22(3):625–39. doi: 10.3758/s13415-021-00967-w (PMC9090867; doi:10.3758/s13415-021-00967-w)
Supplement: Supplementary file 1 — (DOCX 28 kb) [file 13415_2021_967_MOESM1_ESM.docx]

Supplementary Table 1. Demographics for the two groups (full behavioural sample).

|  | Autism (N = 24,  13 male)  *M (SD)* | Neurotypical (N = 22, 13 male)  *M (SD)* | T, p-values |
| --- | --- | --- | --- |
| Age (years) | 30.9 (7.2) | 31.4 (8.7) | 0.21, p = .84 |
| AQ | 36.6 (6.7) | 15.3 (7.9) | 9.91, p < .001 |
| WASI | 110.6 (12.3) | 114.2 (13.2) | 0.96, p = .34 |

Note: AQ = Autism Spectrum Quotient, WASI = Wechsler Abbreviated Scale of Intelligence.
